# The pharmacokinetic profile of a novel fixed-dose combination tablet of ibuprofen and paracetamol

**DOI:** 10.1186/1472-6904-10-10

**Published:** 2010-07-05

**Authors:** Trevor Tanner, Sue Aspley, Andrew Munn, Tracy Thomas

**Affiliations:** 1 Simbec Research Ltd, Cardiff Road, Merthyr Tydfil, CF48 4DR, UK; 2 Reckitt Benckiser Healthcare UK Ltd, Dansom Lane, Hull, Yorkshire, HU8 7DS, UK

## Abstract

**Background:**

Ibuprofen and paracetamol differ in their mode of action and related therapeutic effects, suggesting that combined administration may offer improved analgesia. Reported here are the results of two studies on the pharmacokinetic properties of a novel ibuprofen (200 mg) and paracetamol (500 mg) fixed-dose combination tablet.

**Methods:**

Both studies were open-label, randomised studies in healthy volunteers: Study 1 was a four-way crossover, single-dose study; Study 2 was a two-way cross-over, repeat-dose study.

**Results:**

Pharmacokinetic parameters for ibuprofen and paracetamol were similar for the combination and monotherapy tablets (values falling within the 80% to 125% acceptable bioequivalence range) except for the rate of absorption of paracetamol from the combination (t_max_), which was significantly faster compared with monotherapy (median difference 10 minutes; p < 0.05). Mean plasma concentrations of both drugs were higher, earlier, following administration of the combination tablet compared with monotherapy. Mean plasma levels at 10 and 20 minutes were 6.64 μg.mL^-1 ^and 16.81 μg.mL^-1^, respectively, for ibuprofen from the combination, compared with 0.58 μg.mL^-1 ^and 9.00 μg.mL^-1^, respectively, for monotherapy. For paracetamol, mean plasma levels at 10 and 20 minutes were 5.43 μg.mL^-1 ^and 14.54 μg.mL^-1^, respectively, for the combination compared with 0.33 μg.mL^-1 ^and 9.19 μg.mL^-1^, respectively, for monotherapy. The rate of absorption of both ibuprofen and paracetamol was significantly delayed when the combination tablet was administered in the fed versus fasted state; median delay was 25 minutes for ibuprofen (p > 0.05) and 55 minutes for paracetamol (p < 0.001). The pharmacokinetic parameters were comparable irrespective of whether the combination tablet was given twice or three times daily; systemic exposure was, however, approximately 1.4 times greater for both drugs when given three times daily.

**Conclusions:**

Administration of ibuprofen and paracetamol in a fixed-dose combination tablet does not significantly alter the pharmacokinetic profiles of either drug, except for enhancing the rate of paracetamol absorption, offering potential therapeutic benefits in relation to the onset of analgesia. Concentrations of both drugs reached previously reported therapeutic levels when the combination tablet was administrated in the fed or fasted state. Three times daily dosing may offer enhanced therapeutic effect for longer than twice daily dosing.

## Background

Ibuprofen and paracetamol are the most commonly used non-prescription analgesics. These two compounds differ in their mode of action. Ibuprofen is a non-steroidal anti-inflammatory drug (NSAID) that inhibits cyclooxygenase enzymes (COX-1 and COX-2) and subsequent synthesis of prostaglandins and related compounds at peripheral sites within injured tissue [1]. The mode of action of paracetamol is not completely understood, but appears to be related to the inhibition of a sub-class of cyclooxygenase enzyme isoforms in the central nervous system [2].

Both ibuprofen and paracetamol are rapidly absorbed after oral administration, with peak serum concentrations occurring within one to two hours for ibuprofen and between 30 minutes and two hours for paracetamol. Both drugs have plasma half lives of approximately two hours, although in the case of paracetamol this can vary between one and four hours [3]. Ibuprofen is extensively bound to plasma proteins (99%) and extensively metabolised in the liver to two major inactive metabolites that are rapidly and completely excreted by the kidneys. In contrast, paracetamol has low plasma binding (20%) and although also extensively metabolised in the liver to two groups of major metabolites, the sulphate conjugates of these metabolites can accumulate in the event of overdose, due to enzyme saturation. Paracetamol also differs from ibuprofen in that it has no anti-inflammatory actions. Furthermore, paracetamol is observed to provide less effective analgesia than NSAIDs in some indications such as dental pain and sore throat [4,5].

Taken together these differing modes of action and related therapeutic effects suggest that ibuprofen and paracetamol may complement each other and improved analgesia may be obtained using a combination, compared with individual administration. A fixed-dose combination tablet offers the advantages of patient compliance and convenience, although it potentially limits the scope for dosage adjustment. A small study on the pharmacokinetic properties of ibuprofen and paracetamol, when taken concurrently, found no significant change in kinetic parameters of the two drugs [6]. Here we report the findings of two studies on the pharmacokinetic properties of a novel ibuprofen (200 mg) and paracetamol (500 mg) fixed-dose combination tablet. These studies were conducted to investigate the bioavailability, and effect of food on bioavailability, of ibuprofen and paracetamol from this novel single tablet combination and to determine that there was no pharmacokinetic drug-drug interaction when these two drugs are combined in a single tablet.

## Methods

### Study design and objectives

These studies were designed to determine the pharmacokinetic properties of a novel fixed-dose combination tablet of ibuprofen (200 mg) and paracetamol (500 mg). The primary objective of Study 1 was to show that the pharmacokinetic profiles of ibuprofen and paracetamol do not change significantly when administered as a fixed-dose combination tablet compared with monotherapy. The primary objective of Study 2 was to investigate the pharmacokinetic profiles of ibuprofen and paracetamol following twice or three times daily dosing with a fixed-dose combination tablet. The secondary objectives were to investigate the effects of food intake on the single dose pharmacokinetic profiles of the fixed-dose combination tablet (Study 1) and to assess its safety profile in healthy volunteers (Study 1 and 2). The studies were designed in accordance with the Committee for Proprietary Medicinal Products on the investigation of bioavailability and bioequivalence guidelines on fixed combination products [7,8] and US Food and Drug Administration guidance for industry on bioavailability and bioequivalence of oral drugs and food effect [9,10].

Study 1 was an open-label, four-way crossover, randomised, single-dose, single centre, pharmacokinetic study and Study 2 was an open-label, two-way, cross-over, randomised, repeat-dose, single centre, pharmacokinetic study. Both studies were in healthy volunteers and were approved by the South East Wales Research Ethics Committee and conducted in accordance with the Declaration of Helsinki, International Conference on Harmonisation (ICH), Good Clinical Practice (GCP) and applicable regulatory requirements. All participants provided written informed consent.

### Participant selection

The within-subject coefficient of variation for paracetamol maximum plasma concentration (C_max_) was previously estimated to be approximately 25% (unpublished results; Simbec Internal Study Report). Using the method of Diletti [11] a sample size of 24 was calculated to be sufficient to detect a 20% difference between the test and reference formulation, with a power of 80% and an alpha of 5% based on a test versus reference ratio of 1.0. In both studies 26 subjects were enrolled to allow for dropouts.

Subjects were recruited from Simbec Research Ltd (Merthyr Tydfil, Wales, UK) volunteer database and were required to be healthy, between 18 and 75 years of age and have a body mass index (BMI) of 20-27 kg/m^2^. Interested subjects were screened for suitability; screening included recording of demographic data and vital signs, physical examination, medical history, current medical status, prior medication (within 14 days of screening) and concomitant medication. Individuals were not considered suitable if they had a significant history of disease, metabolism disorder or allergy relating to ibuprofen, aspirin, other NSAID or paracetamol, or if they had a history of peptic or duodenal ulcers, gastrointestinal bleeding, frequent dyspepsia or migraine. Other exclusion criteria included a history of drug or alcohol abuse, positive HIV status or known risk factors for AIDS, participation in another trial within 12 weeks of screening, being a current smoker (or having smoked within the previous six months), taking a prescribed drug within 14 days of enrolment or an over the counter drug within seven days (excluding the contraceptive pill or hormone replacement therapy).

A blood sample was taken for haematology, biochemistry and virology screening, together with serum pregnancy test for female subjects. In addition, a urine sample was taken for urinalysis and alcohol and drug testing.

### Treatment and study procedures

In Study 1, subjects were randomised to receive two ibuprofen 200 mg tablets following an overnight fast, two paracetamol 500 mg tablets following an overnight fast or two fixed-dose combination tablets (ibuprofen 200 mg plus paracetamol 500 mg) following an overnight fast, or a standard meal. Treatment was repeated on four occasions, with a washout period of between three and seven days between treatments. Blood samples were taken for pharmacokinetic sampling prior to dosing and post dose at 5, 10, 20, 30 and 40 minutes and 1, 1.25, 1.5, 2, 3, 6, 9 and 12 hours.

In Study 2, subjects were randomised to receive either twice daily (12 hourly) or three times daily (8 hourly) dosing of two fixed-dose combination tablets (ibuprofen 200 mg plus paracetamol 500 mg), for three days on two separate occasions. Subjects randomised to receive twice daily dosing received a total of 800 mg of ibuprofen and 2000 mg of paracetamol per day, and subjects on the three times daily dosing schedule received a total of 1200 mg of ibuprofen and 3000 mg of paracetamol per day. A washout period of between three and seven days was allowed between treatments. Subjects attended the treatment centre the night before treatment (Day 1) and fasted overnight for approximately 10 hours. They then received their first dose at about 07.00 on Day 2 and, in the case of twice daily dosing, their second dose at about 19.00, two hours after dinner. For three times daily dosing, subjects received their second dose at about 15.00, three hours after lunch, and their third dose at about 23.00, two hours after receiving an evening snack. On Days 2 and 4, blood samples were taken for pharmacokinetic sampling prior to dosing and post Dose 1 at 10, 20, 40 and 60 minutes and 1.5, 2, 3, 6, 8 and 12 hours. Further samples were taken prior to administration of Dose 3 on Day 2, prior to administration of all three doses on Day 3 and at 16, 17, 18, 20 and 24 hours post Dose 1 on Day 4.

### Laboratory procedures

All haematology, biochemistry and urinary analyses were conducted using standard methodologies, within a single laboratory. Plasma concentrations of ibuprofen and paracetamol were analysed using liquid chromatography-mass spectrometry procedures, which were fully validated and developed from methods previously used by Simbec Research Ltd (Simbec Data on File).

Paracetamol plasma concentrations were analysed using a liquid:liquid extraction (3:2; diethylether:dichloromethane mixture) followed by LC-MS-MS analysis using a Zorbax SB-C18 column (Agilent Technologies, Santa Clara, USA). The mobile phase consisted of 500 mL methanol, 500 mL water and 5 mL formic acid with an isocratic flow rate of 0.6 mL.min^-1 ^(based on a previously published method) [12].

Ibuprofen plasma concentrations were analysed using a protein precipitation extraction (acetonitrile containing internal standard flurbiprofen) and by LC-MS analysis using a Thermo Electron HyPurity^® ^C18 column (Thermo Electron Corporation, Massachusetts, USA). The mobile phase consisted of 49.95% (v:v) of 10 mM ammonium acetate, 49.95% (v:v) of acetonitrile and 0.10% (v:v) of glacial acetic acid with an isocratic flow rate of 0.21 mL.min^-1^.

### Pharmacokinetic and safety endpoints

Pharmacokinetic parameters for ibuprofen and paracetamol were calculated using WinNonLin Pro^® ^version 5.0.1 (Pharsight Corporation, California, USA). In Study 1, the pharmacokinetic parameters assessed were maximum concentration (C_max_); time to first occurrence of the maximum plasma concentration (t_max_); plasma concentration half life (t_1/2_); elimination rate constant (K_el_), calculated from the slope of the terminal portion of the plasma profile calculated by least-square regression of log.(concentration) against time; area under the plasma concentration-time curve to the last measurable plasma concentration (AUC_0-t_), calculated by the linear trapezoidal rule; and area under the curve calculated by linear trapezoidal rule to the last measurable plasma concentration (C_p_), with additional area calculated from C_p_/K_el _(AUC_0-inf_).

On Day 2 in Study 2, the pharmacokinetic parameters assessed after Dose 1 were C_max_, t_max_, AUC_0-t _and AUC_0-inf_. On Day 4, these same parameters were re-evaluated in addition to the minimum plasma concentration (C_min_), the average plasma concentration (C_av_), fluctuation ([C_max_-C_min_]/C_av_) and the area under the curve calculated by linear trapezoidal rule during a dosage interval in steady state (AUC_tau_).

Safety was assessed in terms of the overall proportion of subjects with adverse events (AEs). Subjects were asked 'Are you experiencing any symptoms or complaints?' at their baseline visit and 'Have you had any symptoms or complaints since you were last asked?' at each treatment visit and specified time points during the study. AEs in response to this questioning or spontaneously reported by subjects were coded using the MedDRA version 9.0 dictionary. All AEs were followed-up and recorded by severity (mild, moderate or severe) and relationship to study medication (definite, probable, possible, unlikely, or none).

### Statistical methods

SAS for Windows 9.1.3 software (SAS Institute Inc., Cary, USA) was used for all statistical calculations. For the calculation of AUCs, the actual rather than nominal sampling time was used in calculations and no imputation methods were used for missing data points.

For Study 1 results, following logarithmic transformation, C_max_, AUC_0-t _and AUC_0-inf _values were subjected to an analysis of variance (ANOVA), including terms for sequence, subject nested within sequence, period and treatment. Contrasts between each pair of treatments (least square [LS] means) were presented together with 90% confidence intervals (CIs) for the difference between treatments constructed using the residual mean square error obtained from the ANOVA. The point and interval estimates were then back transformed to give estimates of the LS geometric mean ratios and their corresponding 90% CI. t_max _was analysed between each pair of treatments using the Wilcoxon matched pairs test. In addition, 95% non-parametric CIs were constructed for the median differences in the t_max _values based on the Hodges-Lehmann estimates.

In Study 2, logarithmically transformed trough values on Days 2, 3 and 4 were used to determine if a steady state had been reached for both treatments. The point estimates were then back-transformed to give estimates of the ratios of the geometric means and the corresponding 95% CI and paired t-tests were also used for each treatment. Following logarithmic transformation, C_max _and AUC_0-t _values on Day 4 were subjected to ANOVA including terms for sequence, subject nested within sequence and period of treatment. Point estimates (using LS) and 90% CIs of the differences between treatments were constructed using the residual mean square error obtained from the ANOVA. The point and interval estimates were then back-transformed to give estimates of the LS geometric mean ratios and their corresponding 90% CIs. In addition, logarithmic AUC_tau _on Day 4 and AUC_0-inf _on Day 2 were subject to an ANOVA (by treatment), including terms for sequence, subject nested within sequence and day. For comparison, point estimates and 90% CIs for the difference between Day 4 and Day 2 were constructed for each treatment using the residual mean square error obtained from the ANOVA. The point and interval estimates were then back-transformed to give estimates of the LS geometric mean ratios and their corresponding 90% CIs.

In both studies the Fisher Exact Test was used to compare the incidence of AEs, in addition to those described as definitely, probably or possibly related to study treatment.

## Results

### Patient enrolment

Forty five subjects were screened for Study 1, of which 27 were enrolled and 25 subjects completed the study. In Study 2, of 33 subjects screened 26 were enrolled on the study. The average age of participants was similar in both studies; 31 years (range 18-57 years) in Study 1 and 33 years (range 20-59 years) in Study 2. The mean BMI and sex distribution were also similar in both studies; mean BMI of 24.5 and 24.4 in Study 1 and 2, respectively, and 16 and 17 subjects were male in Study 1 and 2, respectively. Participants in both studies were current non-smokers and the majority consumed between one and 20 units of alcohol per week.

### Plasma concentration following combination therapy versus monotherapy

The dissolution of the fixed-dose combination tablet has previously been observed to be rapid, with approximately 80% of ibuprofen and paracetamol dissolved within 10 minutes (data not shown). The observed rate and extent of absorption of both ibuprofen and paracetamol from the fixed-dose combination tablet was considered to be bioequivalent to that of monotherapy; however, the rate of absorption of paracetamol was significantly faster for the combination tablet compared with paracetamol monotherapy. The median t_max _for ibuprofen was 75 minutes for both the combination tablet and monotherapy (Table 1). In contrast, the median t_max _for paracetamol was 30 minutes for the combination tablet and 40 minutes for monotherapy, giving a statistically significant median difference in t_max _of 10 minutes (p < 0.05) in favour of the combination tablet.

**Table 1:** Mean derived pharmacokinetic profiles of ibuprofen and paracetamol after administration of the fixed-dose combination tablet compared with monotherapy (Study 1)

	Ibuprofen (combination)	Ibuprofen (monotherapy)	Paracetamol (combination)	Paracetamol (monotherapy)
**n**	25	25	25	25

**t**_**max**_**, hrs **(median difference [minutes], 95% CI)	1.25^a^ (7.5, -15.0-37.5)	1.25^a^	0.50^a^ (-15.0, -30.0-0.0)	0.67^a^

**t**_**1/2**_**, hrs**	1.95	1.97	2.83	2.66

**C**_**max**_**, μg.mL**^**-1**^	32.04	30.89	18.48	17.49

LS geometric means (ratio test/reference [%], 90% CI)	31.46 (104.29, 95.90-113.41)	30.16	17.58 (104.14, 91.32-118.76)	16.88

**AUC**_**0-t**_**, μg.mL**^**-1**^**.hr**	118.32	111.59	51.69	49.47

LS geometric means (ratio test/reference [%], 90% CI)	116.51 (107.08, 103.20-111.11)	108.80	50.27 (104.10, 100.08-108.29)	48.29

**AUC**_**0-inf**_**, μg.mL**^**-1**^**.hr**	120.92	114.09	54.49	51.85

LS geometric means (ratio test/reference [%], 90% CI)	118.82 (106.99, 103.26-110.85)	111.06	52.95 (104.6, 100.56-108.82)	50.62

AUC_0-t_: area under the curve calculated by linear trapezoidal rule to the last measurable plasma concentration; AUC_0-inf_: area under the curve calculated by linear trapezoidal rule to the last measurable plasma concentration (C_p_) with additional area calculated from C_p_/Kel (elimination rate constant); C_max_: maximum plasma concentration; hr(s): hour(s); LS: least square; t_max_: the time to occurrence of the maximum plasma concentration; t_1/2_: plasma concentration half life. ^a^Median values.

**Figure 1:**
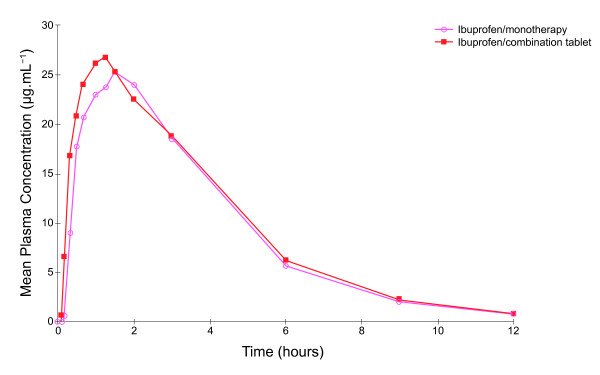
**Mean plasma ibuprofen concentration time curves after administration of ibuprofen monotherapy and ibuprofen-paracetamol fixed-dose combination tablets (Study 1)**.

**Figure 2:**
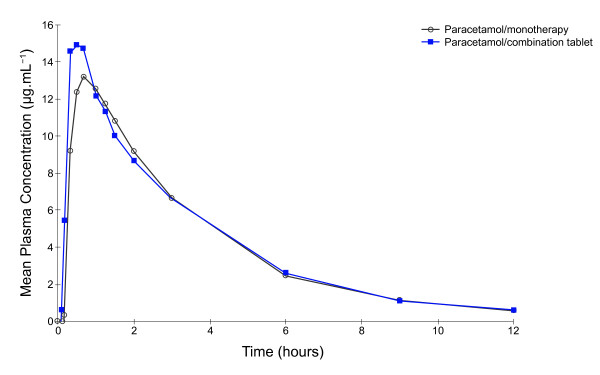
**Mean plasma paracetamol concentration time curves after administration of paracetamol monotherapy and ibuprofen-paracetamol fixed-dose combination tablets (Study 1)**.

Mean plasma concentrations of both ibuprofen and paracetamol were higher, earlier, following administration of the fixed-dose combination tablet compared with administration of the corresponding monotherapy (Table 1, Figure 1 and Figure 2). In subjects receiving the combination tablet the mean ibuprofen plasma levels were 6.64 μg.mL^-1 ^and 16.81 μg.mL^-1 ^at 10 and 20 minutes, respectively, compared with levels of 0.58 μg.mL^-1 ^and 9.00 μg.mL^-1^, respectively, in subjects receiving ibuprofen monotherapy. Similarly, mean plasma concentrations of paracetamol at 10 and 20 minutes in subjects receiving the combination tablet were 5.43 μg.mL^-1 ^and 14.54 μg.mL^-1^, respectively, compared with values of 0.33 μg.mL^-1 ^and 9.19 μg.mL^-1^, respectively, for paracetamol monotherapy.

The pharmacokinetic parameters C_max_, AUC_0-t _and AUC_0-inf _for ibuprofen and paracetamol were similar between the fixed-dose combination and monotherapy tablets (Table 1). The back-transformed 90% CIs for the C_max_, AUC_0-t _and AUC_0-inf _ratios fell within the 80% to 125% acceptable bioequivalence range.

### Fed versus fasted pharmacokinetic profiles

**Figure 3:**
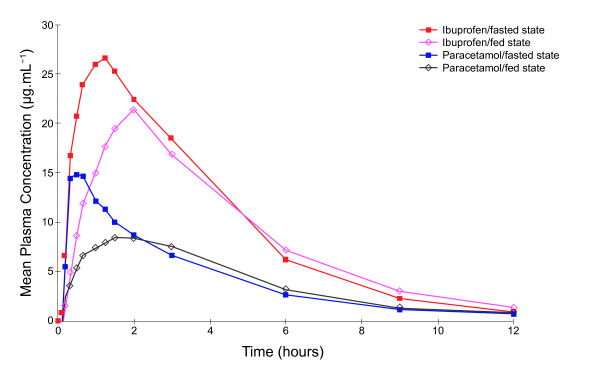
**Mean plasma ibuprofen and paracetamol concentration time curves after administration of ibuprofen-paracetamol fixed-dose combination tablets in the fed and fasted states (Study 1)**.

The absorption of both ibuprofen and paracetamol was significantly delayed when the fixed-dose combination tablet was administrated following a standard meal compared with when it was administered to participants in the fasted state (Table 2, Figure 3). Median delay in rate of absorption was 25 minutes for ibuprofen (p > 0.05) and 55 minutes for paracetamol (p < 0.001). This delay was associated with a reduced C_max _for both ibuprofen and paracetamol when the combination tablet was administered in the fed state; the back-transformed 90% CIs for the C_max _for ibuprofen and paracetamol were outside of the bioequivalence acceptance range of 80% to 125%. In addition, the ibuprofen t_1/2 _was prolonged when the combination tablet was given in the fed versus fasted state, although the paracetamol t_1/2 _did not differ.

**Table 2:** Mean derived pharmacokinetic profiles for ibuprofen and paracetamol after administration of the fixed-dose combination tables in the fed and fasted states (Study 1)

	Ibuprofen		Paracetamol	
	Fed	Fasted	Fed	Fasted
**n**	25	25	25	25

**t**_**max**_**, hrs**	2.00^a^	1.25^a^	1.50^a^	0.50^a^

Median difference [minutes] fed vs fasted, 95% CI	25.0, 0.0-45.0		55.0, 30.0-80.0	

**t**_**1/2**_**, hrs**	2.25	1.95	2.73	2.83

**C**_**max**_**, μg.mL**^**-1**^	24.74	32.04	11.14	18.48

LS geometric mean	24.03	31.46	10.71	17.58

Ratio fed/fasted (%), 90% CI	76.38, 70.25-83.06		60.92, 53.43-69.46	

**AUC**_**0-t**_**, μg.mL**^**-1**^**.hr**	103.91	118.32	46.45	51.69

LS geometric mean	101.6 2	116.51	45.69	50.27

Ratio fed/fasted (%), 90% CI	87.22, 84.06-90.49		90.89, 87.38-94.54	

**AUC**_**0-inf**_**, μg.mL**^**-1**^**.hr**	109.03	120.92	49.51	54.49

LS geometric mean	106.04	118.82	48.72	52.95

Ratio fed/fasted (%), 90% CI	89.25, 86.14-92.46		92.01, 88.45-95.71	

**Kel**	0.328	0.361	0.258	0.251

AUC_0-inf_: area under the curve calculated by linear trapezoidal rule to the last measurable plasma concentration (Cp) with additional area calculated from Cp/Kel; AUC_0-t_: area under the curve calculated by linear trapexoidal rule to the last measurable plasma concentration; C_max_: maximum plasma concentration; Kel: elimination rate constant; t_max_: time of maximum plasma concentration; hr(s): hour(s); LS: least square; sd: standard deviation. ^a^Median values.

In the fed state, the extent of absorption, as indicated by AUC_0-t_, of both ibuprofen and paracetamol from the fixed-dose combination tablet was slightly less compared with the fasted state (Table 2), although the back-transformed 90% CIs for the AUC_0-t_, along with AUC_0-inf_, ratios fell within the bioequivalence acceptance range.

### Pharmacokinetic profiles of twice versus three times daily dosing

In Study 2 the pharmacokinetic parameters for paracetamol, from the fixed-dose combination tablet, were similar following administration of the first dose of both the twice and three times daily dosing regime. However, for ibuprofen a slightly shorter t_max _and higher C_max _values were observed for the three compared with twice times daily dosing schedule (Table 3).

**Table 3:** Mean derived comparison of the single-dose pharmacokinetic parameters in subjects allocated to the twice or three times daily dosing regimen (Study 2; Day 2)

	Ibuprofen		Paracetamol	
	Twice daily	Three times daily	Twice daily	Three times daily
**n**	26	26	26	26

**t**_**max**_**, hrs**	1.75^a^	1.25^a^	0.67^a^	0.67^a^
**C**_**max**_**, μg.mL^-1 ^(sd)**	32.53 (6.15)	36.66 (8.14)	14.74 (5.76)	15.86 (4.76)
**AUC**_**0-t**_**, μg.mL**^**-1**^**.hr (sd)**	128.29 (23.60)	124.01 (23.35)	46.98 (12.42)	47.58 (12.79)

**AUC**_**0-inf**_**, μg.mL**^**-1**^**.hr (sd)**	130.67 (24.61)	132.27 (25.54)	49.23 (12.99)	52.49 (14.06)

AUC_0-inf_: area under the curve calculated by linear trapezoidal rule to the last measurable plasma concentration (Cp) with additional area calculated from Cp/Kel (elimination rate constant); AUC_0-t_: area under the curve calculated by linear trapexoidal rule to the last measurable plasma concentration; C_max_: maximum plasma concentration; hr(s): hour(s); t_max_: time of maximum plasma concentration; t_1/2_: plasma concentration half life; sd: standard deviation. ^a^Median values.

Following three days of treatment (on Day 4) the mean plasma exposures after multiple dosing (AUC_tau_) (Table 4) were comparable to those observed after a single dose (AUC_0-inf_) (Table 1). The LS geometric mean ratios for ibuprofen were 90.28 and 86.50 following twice and three times daily dosing, respectively, and 105.60 and 97.79, respectively, for paracetamol; the 90% CI fell within 80 to 110%. Furthermore, irrespective of whether the combination tablet was given twice or three times daily, the C_max _values observed for ibuprofen and paracetamol were comparable, with LS geometric mean ratios of 100.58 and 98.51, respectively; and the 90% CI fell within the bioequivalence acceptance range of 80 to 125%. The systemic exposure, indicated by AUC_0-t_, was approximately 1.4 times greater for both ibuprofen and paracetamol when the fixed-dose combination tablet was given three times rather than twice a day, reflecting the additional treatment dose.

**Table 4:** Comparison of the mean-derived pharmacokinetic parameters for ibuprofen and paracetamol after administration of the fixed-dose combination tablets, twice or three times daily for 3 days (Study 2; Day 4)

	Ibuprofen		Paracetamol	
	Twice daily	Three times daily	Twice daily	Three times daily
**n**	26	26	26	26

**t**_**max**_**, hrs**	1.50^a^	1.50^a^	0.67^a^	0.67^a^

**C**_**max**_**, μg.mL**^**-1 **^**(sd)**	33.4 (6.12)	33.55 (7.32)	16.09 (5.14)	15.87 (5.26)

**C**_**min**_**, μg,mL**^**-1 **^**(sd)**	0.72 (0.42)	2.64 (1.24)	0.74 (0.25)	1.87 (0.79)

**C**_**av**_**, μg.mL**^**-1 **^**(sd)**	9.61 (1.96)	13.69 (2.73)	4.07 (1.05)	5.86 (1.68)

**Fluctuation (sd)**	3.44 (0.65)	2.26 (0.50)	3.83 (1.02)	2.47 (0.83)
**(C**_**max**_**-C**_**min**_**)/C**_**av**_				

**Swing (sd)** **(C**_**max**_**-C**_**min**_**)/C**_**min**_	62.47 (40.28)	14.90 (8.72)	22.81 (10.05)	8.73 (4.78)

**AUC**_**0-t**_, **μg.mL**^**-1**^**.hr (sd)**	230.73 (47.00)	328.60 (65.71)	97.67 (25.13)	140.80 (40.30)

**AUC**_**tau**_, **μg.mL**^**-1**^**.hr (sd)**	118.12 (24.23)	114.26 (22.77)	51.72 (12.89)	50.74 (13.29)

AUC_tau_: area under the plasma concentration-time curve for a dosing interval; AUC_0-t_: area under the curve calculated by linear trapezoidal rule to the last measurable plasma concentration; C_av_: average plasma concentration; C_max_: maximum plasma concentration; C_min_: minimum plasma concentration; hr(s): hour(s); t_max_: time of maximum plasma concentration; sd: standard deviation. ^a^Median values.

**Figure 4:**
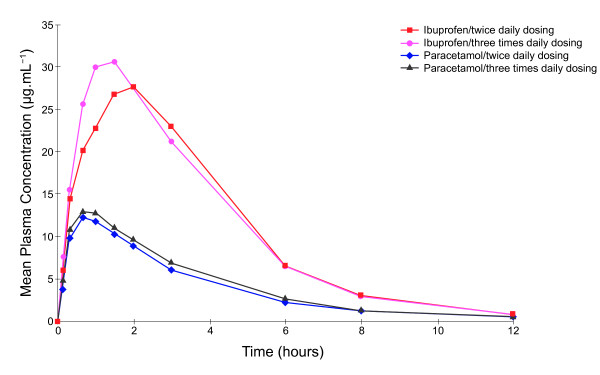
**Mean plasma ibuprofen and paracetamol concentrations time curves following administration of the first dose in subjects allocated to receive twice or three times daily dosing with ibuprofen-paracetamol fixed-dose combination tablets (Study 2; Day 2)**.

On Day 4, the plasma concentrations of both ibuprofen and paracetamol pre-dose and 24 hours post-dose were higher for subjects receiving three times daily dosing compared with those receiving twice daily dosing (Figure 4). Overall, however, the mean plasma levels achieved for both ibuprofen and paracetamol were similar for the two different dosing regimes. Day 4 C_max _and t_max _values for ibuprofen and paracetamol were also similar for the different dosing groups. Both ibuprofen and paracetamol C_min _and C_av _values were, however, greater with three times daily dosing; therefore, less fluctuation and swing was observed compared with twice daily dosing. In addition, AUC_0-t _values for both ibuprofen and paracetamol were higher following three times daily dosing but values for AUC_tau _were similar between the dosing regimes.

**Figure 5:**
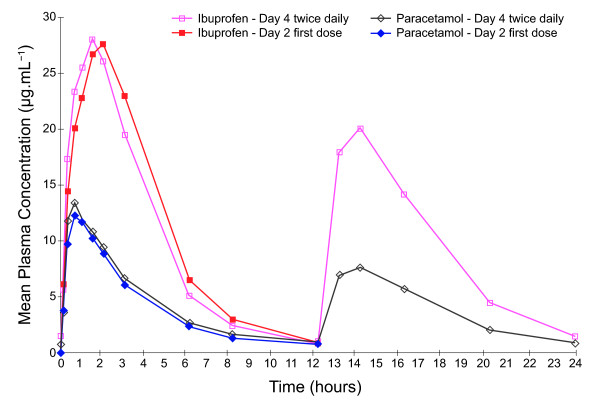
**Comparison of mean plasma ibuprofen and paracetamol concentration time curves after administration of the first dose (on Day 2) and following three days of treatment (Day 4) with ibuprofen-paracetamol fixed-dose combination tablets at twice daily dosing**.

**Figure 6:**
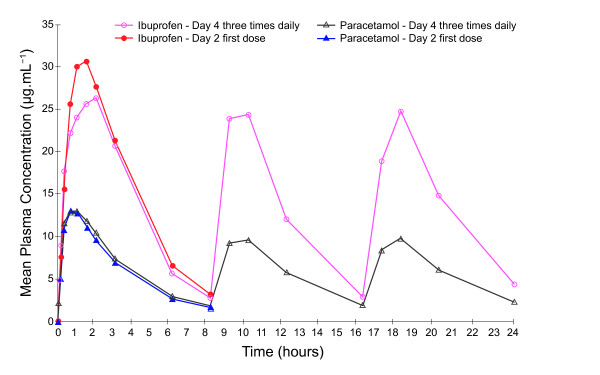
**Comparison of mean plasma ibuprofen and paracetamol concentration time curves after administration of the first dose (on Day 2) and following three days of treatment (Day 4) with ibuprofen-paracetamol fixed-dose combination tablets at three times days daily dosing**.

Analysis of ratios of trough values on Days 2, 3 and 4 found that a steady state was reached for both ibuprofen and paracetamol and no accumulation of either ibuprofen or paracetamol was evident on Day 4. Variation in mean trough concentrations was observed across the study days; mean trough values for paracetamol were lower on the first day of dosing compared with later study days, and trough values observed for ibuprofen and paracetamol were higher in the morning compared with those seen in the afternoon and evening. Thus some statistical differences in the comparison of trough values were observed; LS geometric mean ratio for zero hours, Day 4, versus 12 hours, Day 3, following ibuprofen twice daily dosing, was 1.78 (95% CI 1.47-2.16; p < 0.0001, Figure 5); LS geometric mean ratio for zero hours, Day 4, versus 16 hours, Day 3, for ibuprofen following three times daily dosing was 1.38 (95% CI 1.19-1.61; p < 0.0001, Figure 6). Comparison of trough levels obtained at the same time of day did not show any significant differences between dosing regimes.

### Safety results

No significant changes in haematological or biochemical values were observed during the course of either study and no clinically significant safety issues were identified. There were no serious AEs and all non-serious events were categorised as mild or moderate. In Study 2 there were four AEs recorded in three subjects that were considered possibly or probably related to study medication: a rash on the left arm and trunk lasting between 11 and 21 hours; heartburn, resolving after 1.1 days; and cold like symptoms, resolving after 12.7 hours.

## Discussion

Ibuprofen and paracetamol are rapidly released from this novel fixed-dose combination tablet containing 200 mg of ibuprofen and 500 mg of paracetamol. The results observed in Study 1 show that concurrent administration of ibuprofen and paracetamol in this novel fixed-dose combination tablet did not significantly alter the rate and extent of absorption of ibuprofen or the extent of paracetamol absorption compared with either agent administered alone. The rate of paracetamol absorption was, however, significantly enhanced with the combination tablet compared with paracetamol monotherapy. A previously published repeat-dose study on the pharmacokinetic profiles of ibuprofen (400 mg) and paracetamol (650 mg) showed a shorter, but not significant, t_max _for paracetamol co-administered with ibuprofen (48 minutes) compared with paracetamol monotherapy (54 minutes) [6]. The 10 minute median decrease in t_max _observed in this study may be due either to the comparatively larger dose of paracetamol used or to formulation characteristics and the fact that the fixed-dose combination tablet is very efficient at dissolution.

When the fixed-dose combination tablet was given in the fed state, the rate of absorption of both ibuprofen and paracetamol was delayed and this effect was particularly pronounced for paracetamol; although, the C_max _value for paracetamol remained within the range of therapeutic plasma values considered effective [13]. This observed delay is consistent with previously published studies of ibuprofen and paracetamol monotherapy [13,14] and is expected as the absorption of both drugs takes place in the small intestine, and paracetamol absorption is known to be dependent on gastric emptying [15]. Despite this slight delay in absorption of both drugs, the extent of absorption of ibuprofen and paracetamol was similar when the combination tablet was given in the fed or fasted state. Both ibuprofen and paracetamol 90% CIs for AUC_0-t _and AUC_0-inf _ratios were within the bioequivalence acceptance range of 80 to 125% and, therefore, not considered clinically significant. However, these confidence intervals did not exceed 100% indicating that there may be a non-significant reduction in the extent of absorption.

In Study 2, the multi-dose pharmacokinetics of ibuprofen and paracetamol in the fixed-dose combination tablet were observed to be comparable to the single-dose pharmacokinetics. For ibuprofen, however, there was a slight variation in the rate of absorption, indicating some intra-subject variability. Furthermore, plasma levels of ibuprofen and paracetamol achieved by twice and three times daily dosing were similar, although, as expected, trough concentrations of both drugs were slightly higher following three times relative to twice daily dosing.

A clear relationship between plasma concentrations and degree of measured pain relief has been observed for ibuprofen [16] and for paracetamol [17-19] , although this relationship is not as clear for paracetamol as for ibuprofen, as a lag between plasma concentration and therapeutic affect is observed [20]. Based on these reported levels, the novel combination tablet achieves therapeutic dose levels of ibuprofen and paracetamol for approximately 12 hours per day and 8 hours per day when given three times or twice daily, respectively. Therefore, the three times daily dosing, with less fluctuation and swing in plasma levels, offers greater exposure to clinically effective levels of both ibuprofen and paracetamol, which may be associated with greater therapeutic benefit.

Ibuprofen and paracetamol are analgesic compounds commonly used for treating mild to moderate pain. For the relief of more severe pain, combination analgesia is often recommended, as the combination of analgesics with different modes of action has the potential to offer enhanced pain relief with a comparatively lower dose of each analgesic and corresponding reduced side effects [21]. In both studies this novel ibuprofen and paracetamol fixed-dose combination tablet was well tolerated. The incidence of AEs was low, none of which were considered definitely associated with study medication.

## Conclusion

The combination of ibuprofen and paracetamol in a fixed-dose tablet does not significantly alter the pharmacokinetic profiles of either drug alone, although the rate of paracetamol absorption is enhanced, offering potential therapeutic benefits in relation to the onset of analgesia. Concentrations of both ibuprofen and paracetamol reach levels required for therapeutic effect when the fixed combination formulation is administrated either in the fed or fasted state. The multi-dose pharmacokinetics of the fixed-dose combination tablet are comparable to the single-dose pharmacokinetics and three times daily dosing may offer enhanced therapeutic effect for longer than twice daily dosing.

## Competing interests

Sue Aspley is an employee of Reckitt Benckiser Healthcare International Ltd. Trevor Tanner, Andrew Munn and Tracy Thomas declare that they have no competing interests.

## Authors' contributions

TTa helped design the studies, derived the pharmacokinetic parameters and reviewed the statistical analysis. TTh and AM were involved in the technical undertaking of this study; TTh was responsible for paracetamol bioanalysis and AM for ibuprofen bioanalysis. SA participated in the design of the studies, wrote the protocols and managed the conduct and reporting of the study on behalf of the sponsor. All authors contributed to the content and review of the manuscript and read and approved the final manuscript.

## Authors' information

Trevor Tanner is Scientific Director at Simbec Research (Merthyr Tydfil, Wales, UK). Tracy Thomas and Andrew Munn are Principal Scientists in the Bioanalytical Unit at Simbec Research. Sue Aspley is an employee of Reckitt Benckiser Healthcare International Ltd.

## Pre-publication history

The pre-publication history for this paper can be accessed here:

http://www.biomedcentral.com/1472-6904/10/10/prepub
